# Supplementary material for: Determination of Pesticide Residues in Fresh Fruits in the Serbian Market by LC-MS/MS
Source: Foods. 2025 May 21;14(10):1828. doi: 10.3390/foods14101828 (PMC12111384; doi:10.3390/foods14101828)
Supplement: Supplementary file 1 [file foods-14-01828-s001.zip › foods-3650512-supplementary.pdf]

Supplementary material S1

Table S1. Summary of studies dealing with detection of pesticide residue in fresh fruits/food of plant origin.

Reference	Country of origin	Commodity (fresh)	No. of samples	% of samples without detectable residues (< LOQ)	% of samples with residues at or above the LOQ	% of samples with residues above the MRL
Abdallah et al. [1]	Saudi Arabia	Date	200	82	18	7.5 (MRLs in EU); some samples contained pesticide residues (carbofuran) that are banned for use in the Saudi Arabia
Abou Zeid et al. [2]	Lebanon	Loquat	128	50.78	49.22	39.84 (MRLs in EU); some samples contained pesticide residues that are banned or unauthorised for use in the Lebanon
Ahmed et al. [3]	Egypt	Date	257	45.92	54.08	25.29 (MRLs in Codex/EU)
Al-Antary et al. [4]	Jordan	Fruit (local samples)	94	82.98	17.02	2.13 (MRLs in Jordan/Codex)
		Fruit (imported samples)	202	78.71	21.29	0 (MRLs in Jordan/Codex)
		Vegetable (local samples)	306	92.81	7.19	0.33 (MRLs in Jordan/Codex)
		Vegetable (imported samples)	198	98.99	1.01	0 (MRLs in Jordan/Codex)
Al-Antary et al. [5]	Jordan	Fruit and vegetable	400 (local samples)	95.75	4.25	0.25 (MRLs in Jordan/Codex)
			400 (imported samples)	94.25	5.75	0.25 (MRLs in Jordan/Codex)

Al-Antary et al. [6]	Jordan	Fruit and vegetable	240 (local samples)	87.08	12.92	5.83 (MRLs in Jordan/Codex)
			400 (imported samples)	98.0	2.0	0.75 (MRLs in Jordan/Codex)
Algharibeh and AlFararjeh [7]	Jordan	Fruit and vegetable	158	46	54	22 (MRLs in EU); some samples contained pesticide residues (propargite) that are banned for use in the Jordan/EU
Al-Nasir et al. [8]	Jordan	Citrus fruit and vegetable	40	NM	NM	NM (MRLs in EU); in some samples pesticide residues were above MRLs in EU
Al-Shamary et al. [9]	Qatar	Fruit and vegetable	127	36.8	73.2	62.2 (organochlorines pesticides, MRLs in Codex)
Andraščíková et al. [10]	Portugal	Orange	11	9.1	90.9	36.4 (MRLs in EU); some samples contained pesticide residues (atrazine) that are banned for use in the Portugal
Aslantas et al. [11]	Turkey	Lemon	100	57	43	29 (MRLs in EU); some samples contained pesticide residues (chlorpyrifos-methyl, chlorpyrifos) that are banned for use in the Turkey/EU
Badr et al. [12]	Egypt	Fruit and vegetable	45	NM	NM	NM (MRLs in EU/Codex/FDA/EPA); in some samples pesticide

						residues were above MRLs in Egypt
Bakırcı et al. [13]	Turkey	Fruit	573	40	60	8.4 (MRLs in Turkey)
		Vegetable	850	51	49	9.8 (MRLs in Turkey)
Balkan and Karaağaçlı [14]	Turkey	Tropical fruit	48	58.3	41.7	0 (MRLs in EU)
Baša Česnik et al. [15]	Croatia and Slovenia	White grape	12	41.7	58.3	0 (MRLs in EU)
Baša Česnik et al. [16]	Slovenia	Red grape	73	8.2	91.8	0 (MRLs in EU)
Bempah et al. [17]	Ghana	Fruit and vegetable	350	37.5	62.5	19.0 (MRLs in EU)
Bempah and Donkor [18]	Ghana	Fruit	320	12.8	87.3	38.6 (MRLs in Codex)
Berrada et al. [19]	Spain	Fruit	429	73	27	3.7 (MRLs in Spain/EU)
Bouagga et al. [20]	Tunis	Table grape	64	0	100	94 (MRLs in EU); some samples contained pesticide residues that are banned for use in the Tunis
Chen et al. [21]	China	Fruit and vegetable	3,009	62.3	37.7	11.7 (MRLs in China)
Chen et al. [22]	China	Wolfberry	50	0	100	0 (MRLs in China/EU)
Chen et al. [23]	China	Strawberry	40	NM	NM	0 (tetraconazole and kresoxim-methyl, MRLs in China)
Choubbane et al. [24]	Morocco	Fruit and vegetable	51	31	69	0 (MRLs in EU); some samples contained pesticide residues that are banned for use in the Morocco
Chu et al. [25]	China	Strawberry	440	60.68	39.32	0 (MRLs in China)
Čuš et al. [26]	Slovenia	Grape	NM	NM	NM	NM (MRLs in EU); in some samples

						pesticide residues (phosalone) were above MRLs in EU
Danis et al. [27]	Greece	Peach	1,150	66.6	33.3	0 (MRLs in EU)
de Oliveira Mozzaquatro et al. [28]	Brazil	Passion fruit	55	40	60	0 (MRLs in Brazil); some samples contained pesticide residues that are unauthorised for use in the Brazil
Du et al. [29]	China	Grape	54	NM	NM	0 (MRLs in China/EU/Codex)
Dülger and Tiryaki [30]	Turkey	Peach and nectarine	72	NM	NM	0 (MRLs in EU)
Eissa et al. [31]	Egypt	Green grape	32	18.75	81.25	21.88 (MRLs in EU/Codex); some samples contained pesticide residues that are unregistered for use in the Egypt
El Din et al. [32]	Egypt	Grape	24	0	100	100 (organophosphorus pesticides and carbamates, MRLs in EU)
El Hawari et al. [33]	Lebanon	Apple	212	23	77	61 (MRLs in EU)
El-Mageed et al. [34]	United Arab Emirates	Fruit	5,381	61.96	38.04	5.43 (MRLs in EU/Codex); some samples contained pesticide residues that are banned or unauthorised for use in the United Arab Emirates
		Vegetable	4,343	65.43	34.57	7.52 (MRLs in EU/Codex); some samples contained pesticide residues

						that are banned or unauthorised for use in the United Arab Emirates
Elmi et al. [35]	Iran	Pistachio	20	60	40	0 (MRLs in Iran); some samples contained pesticide residue (acetamiprid) that are unauthorised for use in the Iran
El-Sheikh et al. [36]	Egypt	Fruit	54	39	61	38.9 (MRLs in EU)
		Vegetable	66	33	67	40.7 (MRLs in EU)
El-Sheikh et al. [37]	Egypt	Tomato and strawberry and their products	74	0	100	NM (MRLs in EU); in some samples pesticide residues were above MRLs in EU
Farag et al. [38]	Egypt	Herb, fruit and vegetable	132	45.45	54.55	0.76 (carbendazim, MRLs in Egypt)
Gad Alla et al. [39]	Egypt	Fruit	177	28.2	71.7	17.5 (MRLs in EU); some samples contained pesticide residues that are unauthorised for use in the Egypt
Golge and Kabak [40]	Turkey	Orange	400	92.5	7.5	0 (MRLs in EU/Codex)
Golge and Kabak [41]	Turkey	Table grape	280	40.4	59.6	20.4 (MRLs in EU)
Gondo et al. [42]	Botswana	Fruit and vegetable	83	22	78	13 (MRLs in EU/Codex); some samples contained pesticide residues that are unregistered for use in the Botswana
Hasan et al. [43]	Bangladesh	Fruit	100	60	40	0 (MRLs in Codex)
		Vegetable	100	55	45	0 (MRLs in Codex)

Hjorth et al. [44]	South America	Fruit and vegetable	724	19	80.4	8.4 (MRLs in South America/EU)
Ibrahim et al. [45]	Egypt	Fruit and vegetable	175	20	80	42 (MRLs in Codex)
Jallow et al. [46]	Kuwait	Fruit and vegetable	150	42	58	21 (MRLs in Codex); some samples contained pesticide residues (aldrin) that are banned for use in the Kuwait
Jardim and Caldas [47]	Brazil	Fruit, vegetable, rice, and bean	13,556	51.7	48.3	2.7 (MRLs in Brazil); some samples contained pesticide residues that are unauthorized for use in the Brazil
Jardim et al. [48]	Brazil	Cashew apple, guava, kaki, peach and pulp	238	~ 30	~70	0 (MRLs in Brazil); some samples contained pesticide residues that are unauthorized for use in the Brazil
Jeong et al. [49]	South Korea	Omija fruit and juice	320	NM	NM	NM (MRLs in South Korea); some samples contained pesticide residues that are unauthorised for use in the South Korea
Kim et al. [50]	Korea	Fruit, vegetable, rice and soybean	232	70.3	29.7	0.9 (MRLs in Korea)
Knežević et al. [51]	Croatia	Fruit and vegetable	866	66.2	33.8	5.3 (MRLs in EU)
Kowalska et al. [52]	Poland	Apple and selected citrus fruit	26	NM	NM	NM (MRLs in EU); in some samples pesticide residues

						were above MRLs in EU
Kuang et al. [53]	China	Litchi	150	29.3	70.7	NM (MRLs in China); in some samples pesticide residues (carbofuran) were above MRLs in China
Latif et al. [54]	Pakistan	Fruit	131	59.5	40.5	2.3 (MRLs in Codex)
Li et al. [55]	China	Strawberry	245	73.9	26.1	1.63 (MRLs in China)
Li et al. [56]	China	Peach	312	7.7	92.3	3.2 (MRLs in China); some samples contained pesticide residues that are banned for use in the China
Li et al. [57]	China	Pear	310	6.8	93.2	2.6 (MRLs in China)
Li et al. [58]	China	Citrus fruit	2,922	14	86	3.8 (MRLs in China); some samples contained pesticide residues that are banned for use in the China
Liang et al. [59]	China	Fruit	10,551	99.8	0.2	0.05 (fipronil, MRLs in China)
		Vegetable	19,966	99.2	0.8	0.2 (fipronil, MRLs in China)
Likudis et al. [60]	Greece	Apple	80	6.3	93.7	2.5 (parathion-methyl, MRLs in EU); some samples contained pesticide residue (flufenoxuron) that

						are banned for use in the Greece
Liu et al. [61]	China	Fruit	68	4.41	95.59	1.47 (prochloraz, MRLs in China)<br>27.94 (MRLs in EU)
Liu et al. [62]	China	Persimmon	22	63.6	36.4	4.5 (MRLs in China)
		Jujube	24	29.2	70.8	25.0 (MRLs in China)
Liu et al. [63]	China	Nut	44	65.9	34.1	15.9 (MRLs in China)<br>20.5 (MRLs in EU)
Łozowicka et al. [64]	Poland	Raspberry	128	48.4	51.6	21.1 (MRLs in Poland/EU); some samples contained pesticide residues that are unauthorized for use in the Poland
Łozowicka et al. [65]	Poland	Raspberry	184	42.4	57.6	29.3 (EU MRLs); some samples contained pesticide residues that are unauthorized for use in the Poland
Łozowicka et al. [66]	Poland	Fruit	392	48.2	51.8	5.9 (MRLs in EU); some samples contained pesticide residues that are unauthorised for use in the Poland
Łozowicka et al. [67]	Poland	Apple	212	34	66	7 (MRLs in Poland/EU); some samples contained pesticide residues that are not recommended for use in the Poland

Lu et al. [68]	USA and China	Fruit and vegetable	122	NM	> 57	NM
Luo et al. [69]	China	Fruit and vegetable	3,307	69.2	30.8	1.0 (MRLs in China)
Mac Loughlin et al. [70]	Argentina	Fruit and vegetable	135	35	65	36.3 (MRLs in Argentina); some samples contained pesticide residues (endosulfan) that are banned for use in the Argentina
Mahdavi et al. [71]	Iran	Apple	25	32	68	4 (iprodione, MRLs in Iran)
		Grape	25	72	28	0 (MRLs in Iran)
Matta et al. [72]	Estonia	Fruit, vegetable, cereal, processed product, baby food and products of animal origin	316 (2008 year)	47	53	1.6 (MRLs in EU)
			397 (2009 year)	52	48	2.3 (MRLs in EU)
			286 (2010 year)	53	47	2.1 (MRLs in EU)
			268 (2011 year)	65	35	0.7 (MRLs in EU)
Matyaszek et al. [73]	Poland	Berry	250	53.6	46.4	4.0 (MRLs in Poland/EU); some samples contained pesticide residues that are banned for use in the Poland
Mebdoua et al. [74]	Algeria	Fruit and vegetable	160	42.5	57.5	12.5 (MRLs in EU/Codex)
Mert et al. [75]	United Kingdom	Fruit and vegetable	25,822	40.2	59.8	4.0 (MRLs in United Kingdom/EU)
Montiel-León et al. [76]	Canada	Fruit and vegetable	133	53	47	0 (MRLs in Canada/EU)
Mutengwe et al. [77]	South Africa	Fruit and vegetable	199	68	32	1 (MRLs in EU); some samples contained pesticide residues that are

						unregistered for use in the South Africa
Mutengwe et al. [78]	South Africa	Fruit and vegetable	37,838	43.54	56.46	0.32 (MRLs in South African); some samples contained pesticide residues that are unregistered for use in the South Africa
Mutengwe et al. [79]	South Africa	Fruit and vegetable	53	67.9	32.1	1.9 (MRLs in South Africa/EU); some samples contained pesticide residues that are banned or unauthorised for use in the South Africa
Mwanja et al. [80]	Zambia	Fruit and vegetable	30	36.7	63.3	10.0 (MRLs in Codex); some samples contained pesticide residues (dichlorvos) that are banned for use in the Zambia
Nasreddine et al. [81]	Lebanon	Foods of plant origin and drinks	1,860	NM	NM	NM (MRLs in EU); in some samples pesticide residues were above MRLs in EU; some samples contained pesticide residues that are banned for use in the Lebanon
Omeje et al. [82]	Nigeria	Fruit and vegetable	60	NM	NM	NM (MRLs in China); in some samples pesticide residues were above MRLs in Codex

Osaili et al. [83]	United Arab Emirates	Fruit	4,513		73.2 (≤MRL)	26.8 (MRLs in EU)
Oshatunberu et al. [84]	Nigeria	Grain	23	NM	NM	NM (MRLs in EU/Codex); some samples pesticide residues (organochlorine pesticides) were above MRLs in EU/Codex; some samples contained pesticide residues that are banned for use in the Nigeria
Park et al. [85]	Korea	Fruit	97	92.8	7.2	1.03 (MRLs in Korea)
		Vegetable	1,049	92.0	8.0	0.95 (MRLs in Korea)
Parveen et al. [86]	Pakistan	Fruit	120	37.5	62.5	22.0 (MRLs in Codex)
Patiño et al. [87]	Colombia	Fruit	47	43	57	44 (MRLs in EU); some samples contained pesticide residues that are not approved for use in the Colombia
		Vegetable	53	51	49	39 (MRLs in EU); some samples contained pesticide residues that are not approved for use in the Colombia
Picó et al. [88]	Saudi Arabia	Fruit	30	0	100	20 (MRLs in EU/Codex)
Pirsaheb et al. [89]	Iran	Apple	50	38	62	NM (MRLs in Codex); in some samples pesticide residues (diazinon and chlorpyrifos)

						were above MRLs in Codex
Poulsen et al. [90]	Denmark	Fruit and vegetable (70%), cereal (15%), samples of animal origin (15%)	17,309	NM	NM	2.6 (most frequently in fruit, MRLs in EU)
Qin et al. [91]	China	Fruit	99/130	57.6	42.4	0 (MRLs in China)
		Vegetable	123	60.7	39.3	1.63 (fungicide, MRLs in China)
Radulović et al. [92]	Serbia	Citrus fruits	76	0	100	28 (MRLs in EU); some samples contained pesticide residues that are unauthorized for use in the EU
Shin et al. [93]	South Korea	Fruit, vegetable and grain	115	82.6	17.4	0.87 (dinotefuran, MRLs in South Korea)
Si et al. [94]	China	Fruit and vegetable	197	5.1	94.9	0 (MRLs in China);
Sivaperumal et al. [95]	India	Fruit and vegetable	286	NM	83.6 (< MRL in EU); 96 (< MRL in India)	16.4 (MRLs in EU); 4.2. (MRL in India)
Skretteberg et al. [96]	Southeast Asia	Fruit and vegetable	721	60	40	12 (MRLs in EU)
Sójka et al. [97]	Poland	Strawberry (fresh and frozen)	121	35.5	64.5	4.13 (MRLs in Poland/EU)
Soydan et al. [98]	Turkey	Fruit and vegetable	3,044	72.5	27.5	11.6 (MRLs in Turkey)
Suárez-Jacobo et al. [99]	Mexico	Orange	100	56	44	11 (MRLs in EU)
Sungur and Tunur [100]	Turkey	Fruit and vegetable	NM	NM	NM	NM (MRLs in Turkey /EU); in some samples pesticide residues were above MRLs in Turkey /EU
Szpyrka et al. [101]	Poland	Fruit	547	49.4	50.6	2.2 (MRLs in Poland/EU)

		Vegetable	479	79.9	20.7	1.3 (MRLs in Poland/EU)
Toptanci et al. [102]	Turkey	Fruit and vegetable	493	48.5	51.5	29.2 (MRLs in Turkey)
Walorczyk et al. [103]	Poland	Organic crop (fruit, vegetable…)	528	95.6	4.4	NM (MRLs in Poland/EU); some samples contained pesticide residues that are banned or unauthorised for use in the EU
Wang et al. [104]	China	Vegetable	74,029	92.3	7.70	0.48 (carbendazim, over limit ratio, MRLs in China)
		Fruit	24,607	73.6	26.4	0.20 (carbendazim, over limit ratio, MRLs in China)
		Mushroom	17,275	88.7	11.3	0.03 (carbendazim, over limit ratio, MRLs in China)
		Cereal	908	92.8	7.20	0.44 (carbendazim, over limit ratio, MRLs in China)
		Tea	470	99.1	0.90	0 (carbendazim, over limit ratio, MRLs in China)
Wang et al. [105]	China	Litchi	268	0	100	19.8 (MRLs in China); some samples contained pesticide residue (imidacloprid) that are unauthorised for use in the China
Witczak et al. [106]	Poland	Fruit (peel and pulp)	9	0	100	0 (MRLs in EU)
		Vegetables (peel and pulp)	10	0	100	NM (MRLs in EU); in some samples pesticide residues (organophosphorus

						pesticides) were above MRLs in EU
Wołejko et al. [107]	Poland	Berry fruit and juice	170	55.3	44.7	14.7 (MRLs in EU); some samples contained pesticide residues that are unauthorized for use in the Poland
Xing et al. [108]	China	Wolfberry	200	16.5	83.5	NM (MRLs in China); in some samples pesticide residues were above MRLs in China
Yang et al. [109]	China	Bayberry	157	36.9	63.1	2.5 (acetamiprid, MRL in China); some samples contained pesticide residues (isocarbophos) that are banned for use in the China
Yang et al. [110]	China	Minor fruit	87	29.9	70.1	67.8 (MRLs in EU); some samples contained pesticide residues that are unauthorized for use in the China
Yang et al. [111]	China	Minor tropical fruits	117	33.3	66.7	NM (MRLs in China); some samples contained pesticide residues that are unauthorized for use in the China
Zhang et al. [112]	China	Kumquat	573	9.8	90.2	9.4 (MRLs in China); some

						samples contained pesticide residues (methidathion and carbofuran) that are banned for use in the China
Zhang et al. [113]	China	Fruit	260	13.3	87.7	5 (MRLs in China) 33.8 (MRLs in EU)
Zhao et al. [114]	China	Apple	120	8.3	91.7	0 (MRLs in China/Codex/EU/Austria/Japan); some samples contained pesticide residues (chlorpyrifos) that are banned for use in the EU and USA

Codex, Codex Alimentarius Commission; LOQ, limit of quantification; MRL, maximum residue level; NM, not mentioned.

References

1. Abdallah, O.I.; Alamer, S.S.; Alrasheed, A.M. Monitoring pesticide residues in dates marketed in Al-Qassim, Saudi Arabia using a QuEChERS methodology and liquid chromatography–tandem mass spectrometry. *Biomed. Chromatogr*. **2018**, e4199.
2. Abou Zeid, M.I.; Awad, M.K.; Melki, K.C.; Jawdah, Y.A.; Jammoul, A.M. Pesticides residues on Loquat: A minor crop in Lebanon. *Food Control* **2021**, *130*, 108297.
3. Ahmed, W.E.H.; Abd El Megeed, M.E.; Dahroug, S.A.; El Marsafy, A.M.H. Monitoring of pesticide residues in dates produced from different areas of Egypt. *J. Environ. Sci*. **2022**, *51*, 93–118.
4. Al-Antary, T.M.; Alawi, M.A.; Al Awamleh, A.M.; Al-Oqlah, K. Pesticides residues in agricultural crops in northern districts of Jordan in 2010/2011. *Fresenius Environ. Bull*. **2018**, *27*, 2427–2431.
5. Al-Antary, T.M.; Alawi, M.A.; Said, M.; Haddad, N. Monitoring of pesticide residues in agricultural crops in southern governorates of Jordan in 2011/2012. *Fresenius Environ. Bull*. **2018**, *27*, 2418–2426.
6. Al-Antary, T.M.; Alawi, M.A.; Shadermah, A.M.; Haddad, NA. Pesticides residues in agricultural crops in southern governorates of Jordan in 2016 and 2017. *Fresenius Environ. Bull*. **2018**, *27*, 6894–6898.
7. Algharibeh, G.R.; Al Fararjeh, M.S. Pesticide residues in fruits and vegetables in Jordan using liquid chromatography/tandem mass spectrometry. *Food Addit. Contam. Part B Surveill*. **2019**, *12*, 65–73.
8. Al-Nasir, F.M.; Jiries, A.G.; Al-Rabadi, G.J.; Alu'datt, M.H.; Tranchant, C.C.; Al-Dalain, S.A.; Alrabadi, N.; Madanat, O.Y.; Al-Dmour, R.S. Determination of pesticide residues in selected citrus fruits and vegetables cultivated in the Jordan Valley. *LWT - Food Sci. Technol*. **2020**, *123*, 109005.
9. Al-Shamary, N.M.; Al-Ghouti, M.A.; Al-Shaikh, I.; Al-Meer, S.H.; Ahmad, T.A. Evaluation of pesticide residues of organochlorine in vegetables and fruits in Qatar: statistical analysis. *Environ. Monit. Assess*. **2016**, *188*, 198.
10. Andraščíková, M.; Hrouzková. S.; Cunha, S.C. Combination of QuEChERS and DLLME for GC-MS determination of pesticide residues in orange samples. *Food Addit. Contam. Part A Chem. Anal. Control Expo. Risk Assess*. **2013**, *30*, 286–297.
11. Aslantas, S.; Golge, O.; González-Curbelo, M.Á.; Kabak, B. Determination of 355 pesticides in lemon and lemon juice by LC-MS/MS and GC-MS/MS. *Foods* **2023**, *12*, 1812.
12. Badr, A.N.; Ahmed, M.B.M.; Amer, M.M.; Thang, V.N.; Fouzy, A.S.M. Pesticides evaluation in Egyptian fruits and vegetables: A safety assessment study. *J. Environ. Sci. Technol*. **2019**, *12*, 81–91.
13. Bakırcı, G.T.; Acay, D.B.Y.; Bakırcı, F.; Ötleş, S. Pesticide residues in fruits and vegetables from the Aegean region, Turkey. *Food Chem*. **2014**, *160*, 379–392.
14. Balkan, T.; Karaağaçlı, H. Determination of 301 pesticide residues in tropical fruits imported to Turkey using LC–MS/MS and GC-MS. *Food Control* **2023**, *147*, 109576.
15. Baša Česnik, H.; Bolta, Š.V.; Bavčar, D.; Radeka, S.; Lisjak, K. Plant protection product residues in white grapes and wines of "Malvasia Istriana" produced in Istria. *Food Addit. Contam. Part B Surveill*. **2016**, *9*, 256–260.
16. Baša Česnik, H.; Bolta, Š.V.; Lisjak, K. Plant protection product residues in red grapes and Teran PTP wine. *Food Addit. Contam. Part B Surveill*. **2015**, *8*, 113–122.
17. Bempah, C.K.; Buah-Kwofie, A.; Denutsui, D.; Asomaning, J.; Tutu, A.O. Monitoring of pesticide residues in fruits and vegetables and related health risk assessment in Kumasi metropolis, Ghana. *Res. J. Environ. Earth Sci*. **2011**, *3*, 761–771.
18. Bempah, C.K.; Donkor, A.K. Pesticide residues in fruits at the market level in Accra Metropolis, Ghana, a preliminary study. *Environ. Monit. Assess*. **2011**, *175*, 551–561.
19. Berrada, H.; Fernández, M.; Ruiz, M.J.; Moltó, J.C.; Mañes, J.; Font, G. Surveillance of pesticide residues in fruits from Valencia during twenty months (2004/05). *Food Control* **2010**, *21*, 36–44.
20. Bouagga, A.; Chaabane, H.; Toumi, K.; Hamdane, A.M.; Nasraoui, B.; Joly, L. Pesticide residues in Tunisian table grapes and associated risk for consumer's health. *Food Addit. Contam. Part B Surveill*. **2019**, *12*, 135–144.
21. Chen, C.; Qian, Y.; Chen, Q.; Tao, C.; Li, C.; Li, Y. Evaluation of pesticide residues in fruits and vegetables from Xiamen, China. *Food Control* **2011**, *22*, 1114–1120.
22. Chen, J.-N.; Lian, Y.-J.; Zhou, Y.-R.; Wang, M.-H.; Zhang, X.-Q.; Wang, J.-H.; Wu, Y.-N.; Wang, M-L. Determination of 107 pesticide residues in wolfberry with acetate-buffered salt extraction and Sin-QuEChERS

nano column purification coupled with ultra performance liquid chromatography tandem mass spectrometry. *Molecules* **2019**, *24*, 2918.
23. Chen, X.; Fan, X.; Ma, Y.; Hu, J. Dissipation behaviour, residue distribution and dietary risk assessment of tetraconazole and kresoxim-methyl in greenhouse strawberry via RRLCQqQ-MS/MS technique. *Ecotoxicol. Environ. Saf*. **2018**, *148*, 799–804.
24. Choubbane, H.; Ouakhssase, A.; Chahid, A.; Taourirte, M.; Aamouche, A. Pesticides in fruits and vegetables from the Souss Massa region, Morocco. *Food Addit. Contam. Part B Surveill*. **2022**, *15*, 79–88.
25. Chu, Y.; Tong, Z.; Dong, X.; Sun, M.N.; Gao, T.C.; Duan, J.S.; Wang, M. Simultaneous determination of 98 pesticide residues in strawberries using UPLC-MS/MS and GC-MS/MS. *Microchem. J*. **2020**, *156*, 104975.
26. Čuš, F.; Baša Česnik, H.; Bolta, Š.V.; Gregorčic, A. Pesticide residues in grapes and during vinification process. *Food Control* **2010**, *21*, 1512–1518.
27. Danis, T.G.; Karagiozoglou, D.T., Tsakiris, I.N.; Alegakis, A.K.; Tsatsakis, AM. Evaluation of pesticides residues in Greek peaches during 2002–2007 after the implementation of integrated crop management. *Food Chem*. **2011**, *126*, 97–103.
28. de Oliveira Mozzaquatro, J.; César, I.A.; Pinheiro, A.E.B.; Caldas, E.D. Pesticide residues analysis in passion fruit and its processed products by LC–MS/MS and GC–MS/MS: Method validation, processing factors and dietary risk assessment. *Food Chem*. **2022**, *375*, 131643.
29. Du, Y.; Wang, Q.; Yang, G.; Han, F. Determination of 43 pesticide residues in intact grape berries (Vitis Vinifera L.) by using an ultrasound-assisted acetonitrile extraction method followed by LC–MS/MS. *Food Control* **2022**, *140*, 109123.
30. Dülger, H.; Tiryaki, O. Investigation of pesticide residues in peach and nectarine sampled from Çanakkale, Turkey, and consumer dietary risk assessment. *Environ. Monit. Assess*. **2021**, *193*, 561.
31. Eissa, F.I.; Helalia, A.A.; Khorshed, M.A.; El-Sisi, M.A. Monitoring of multi-class pesticide residues in green grape and their potential risk for Egyptian consumer. *Nat. Sci*. **2013**, *11*, 11.
32. El Din, A.M.S.; Azab, M.M.; Shalaby, M.A. Pesticide residues monitoring of organophosphorus and carbamates in grapes in three Egyptian governorates. *Arch. Curr. Res. Int*. **2018**, *12*, 1–12.
33. El Hawari, K.; Mokh, S.; Al Iskandarani, M.; Halloum, W.; Jaber, F. Pesticide residues in Lebanese apples and health risk assessment. *Food Addit. Contam. Part B Surveill*. **2019**, *12*, 81–89.
34. El-Mageed, N.M.A.; Abu-Abdoun, I.I.; Janaan, AS. Monitoring of pesticide residues in imported fruits and vegetables in United Arab Emirates during 2019 (2020). *Int. Res. J. Pure Appl. Chem*. **2020**, *21*, 239–260.
35. Elmi, M.; Ghane, T.; Daraei, B.; Eskandari, S.; Mohammadpour, A.; Amirahmadi, M.; Khaneghah, A.M. Monitoring of pesticide residue in pistachio nut samples by LC/MS-MS. *Food Chem*. **2024**, *437*, 137848.
36. El-Sheikh, El-S.A.; Ramadan, M.M.; El-Sobki, A.E.; Shalaby, A.A.; McCoy, M.R.; Hamed, I.A.; Ashour, M.-B.; Hammock, B.D. Pesticide residues in vegetables and fruits from farmer markets and associated dietary risks. *Molecules* **2022**, 27, 8072.
37. El-Sheikh, El-S.A.; Li, D.; Hamed, I.; Ashour, M.-B.; Hammock, B.D. Residue analysis and risk exposure assessment of multiple pesticides in tomato and strawberry and their products from markets. *Foods* **2023**, *12*, 1936.
38. Farag, R.S.; Abdel Latif, M.S.; Abd El-Gawad, A.E.; Dogheim, S.M. Monitoring of pesticide residues in some Egyptian herbs, fruits and vegetables. *Int. Food Res. J*. **2011**, *18*, 659–665.
39. Gad Alla, S.A.; Almaz, M.M.; Thabet, W.M.; Nabil, M.M. Evaluation of pesticide residues in some Egyptian fruits. *Int. J. Environ*. **2015**, *4*, 87–97.
40. Golge, O.; Kabak, B. Determination of 115 pesticide residues in oranges by high-performance liquid chromatography–triple-quadrupole mass spectrometry in combination with QuEChERS method. *J. Food Compos. Anal*. **2015**, *41*, 86–97.
41. Golge, O.; Kabak, B. Pesticide residues in table grapes and exposure assessment. *J. Agric. Food Chem*. **2018**, *66*, 1701–1713.
42. Gondo, T.F.; Kamakama, M.; Oatametse, B.; Samu, T.; Bogopa, J.; Keikotlhaile, B.M. Pesticide residues in fruits and vegetables from the southern part of Botswana. *Food Addit. Contam. Part B Surveill*. **2021**, *14*, 271–280.
43. Hasan, G.M.M.A.; Das, A.K.; Satter, M.A. Human health risk assessment through the detection of organochlorine pesticides in vegetables and fruits from Dhaka, Bangladesh by gas chromatography tandem mass spectrometry (GC-MS/MS). *Curr. Res. Nutr. Food Sci*. **2022**, *10*, 720–732.

44. Hjorth, K.; Johansen, K.; Holen, B.; Andersson, A.; Christensen, H.B.; Siivinen, K.; Toome, M. Pesticide residues in fruits and vegetables from South America – A Nordic project. *Food Control* **2011**, *22*, 1701–1706.
45. Ibrahim, M.A.; Belal, M.H.; Abdallah, I.S.; El-Sawi, S.A.M. Monitoring and risk assessment of pesticide residues in some locally produced vegetables and fruits. *Egypt. J. Chem*. **2022**, *65*, 429–439.
46. Jallow, M.F.A.; Awadh, D.G.; Albaho, M.S.; Devi, V.Y.; Ahmad, N. Monitoring of pesticide residues in commonly used fruits and vegetables in Kuwait. *Int. J. Environ. Res. Public Health*. **2017**, *14*, 833.
47. Jardim, A.N.O.; Caldas, E.D. Brazilian monitoring programs for pesticide residues in food – Results from 2001 to 2010. *Food Control* **2012**, *25*, 607–616.
48. Jardim, A.N.O.; Mello, D.C.; Goes, F.C.S., Junior, E.F.F.; Caldas, E.D. Pesticide residues in cashew apple, guava, kaki and peach: GC–µECD, GC–FPD and LC–MS/MS multiresidue method validation, analysis and cumulative acute risk assessment. *Food Chem*. **2014**, *164*, 195–204.
49. Jeong, H.R.; Lim, S.J.; Cho, J.Y. Monitoring and risk assessment of pesticides in fresh omija (Schizandra chinensis Baillon) fruit and juice. *Food Chem. Toxicol*. **2012**, *50*, 385–389.
50. Kim, J.-Y.; Lee, S.-M.; Lee, H.-J.; Chang, M.-I.; Kang, N.-S.; Kim, N.-S.; Kim, H.; Cho, Y.-J.; Jeong, J.; Kim, M.K.; Rhee, G.-S. Monitoring and risk assessment of pesticide residues for circulated agricultural commodities in Korea-2013. *J. Appl. Biol. Chem*. **2014**, *57*, 235–242.
51. Knežević, Z.; Serdar, M.; Ahel, M. Risk assessment of the intake of pesticides in Croatian diet. *Food Control* **2012**, *23*, 59–65.
52. Kowalska, G.; Pankiewicz, U.; Kowalski, R. Assessment of pesticide content in apples and selected citrus fruits subjected to simple culinary processing. *Appl. Sci*. **2022**, *12*, 1417.
53. Kuang, L.; Xu, G.; Tong, Y.; Li, H.; Zhang, J.; Shen, Y., Cheng, Y. Risk Assessment of Pesticide Residues in Chinese Litchis. *J. Food Prot*. **2022**, *85*, 98–103.
54. Latif, Y.; Sherazi, S.T.H; Bhanger, M.I. Monitoring of pesticide residues in commonly used fruits in Hyderabad region, Pakistan. *Am. J. Anal. Chem*. **2011**, *2*, 46–52.
55. Li, J.; Chen, W.; Deng, K.; Liu, S.; Li, B.; Li, Y. Monitoring and dietary exposure assessment of pesticide residues in strawberry in Beijing, China. *Food Addit. Contam. Part B Surveill*. **2022**, *15*, 98–105.
56. Li, Z.; Nie, J.; Yan, Z.; Cheng, Y.; Lan, F.; Huang, Y.; Chen, Q.; Zhao, X.; Li, A. A monitoring survey and dietary risk assessment for pesticide residues on peaches in China. *Regul. Toxicol. Pharmacol*. **2018**, *97*, 152–162.
57. Li, Z.; Nie, J.; Yan, Z.; Xu, G.; Li, H.; Kuang, L.; Pan, L.; Xie, H.; Wang, C.; Liu, C.; Zhao, X.; Guo, Y. Risk assessment and ranking of pesticide residues in Chinese pears. *J. Integr. Agric*. **2015**, *14*, 2328–2339.
58. Li, Z.; Zhang, Y.; Zhao, Q.; Wang, C.; Cui, Y.; Li, J.; Chen, A.; Liang, G.; Jiao, B. Occurrence, temporal variation, quality and safety assessment of pesticide residues on citrus fruits in China. *Chemosphere* **2020**, *258*, 127381.
59. Liang, S.-x.; Zhao, Z.; Fan, C.-l.; Xu, J.-z.; Li, H.; Chang, Q.-y.; Pang, G.-f. Fipronil residues and risk assessment of Chinese marketed fruits and vegetables: A long-term investigation over 6 years. *Food Control* **2019**, *106*, 106734.
60. Likudis, Z.; Costarelli, V.; Vitoratos, A.; Apostolopoulos, C. Pesticide residues in Greek apples with protected geographical indication or designation of origin. *J. Pestic. Sci*. **2014**, *39*, 29–35.
61. Liu, Y.; Bei, K.; Zheng, W.; Yu, G.; Sun, C. Pesticide residues risk assessment and quality evaluation of four characteristic fruits in Zhejiang Province, China. *Front. Environ. Sci*. **2023**, *11*, 1124094.
62. Liu, Y.; Li, S.; Ni, Z.; Qu, M.; Zhong, D.; Ye, C.; Tang, F. Pesticides in persimmons, jujubes and soil from China: Residue levels, risk assessment and relationship between fruits and soils. *Sci. Total. Environ*. **2016**, *542*, 620–628.
63. Liu, Y.; Shen, D.; Li, S.; Ni, Z.; Ding, M.; Ye, C.; Tang, F. Residue levels and risk assessment of pesticides in nuts of China. *Chemosphere* **2016**, *144*, 645–651.
64. Łozowicka, B.; Kaczyński, P.; Jankowska, M.; Rutkowska, E.; Hrynko, I. Pesticide residues in raspberries (Rubus idaeus L.) and dietary risk assessment. *Food Addit. Contam. Part B Surveill*. **2012**, *5*, 165–171.
65. Łozowicka, B.; Kaczyński, P.; Jankowska, M.; Rutkowska, E.; Hrynko, I.; Paritowa, A. Pesticide residues in raspberries and their risk assessment. *Int. Food Res. J*. **2014**, *21*, 663–672.
66. Łozowicka, B.; Kaczyński, P.; Rutkowska, E.; Jankowska, M.; Hrynko, I. Evaluation of pesticide residues in fruit from Poland and health risk assessment. *Agric. Sci*. **2013**, *4*, 106–111.

67. Łozowicka, B.; Kaczyński, P. Pesticide residues in apples (2005–2010). *Arch. Environ. Prot.* **2011**, *37*, 43–54.
68. Lu, C.; Chang, C.-H.; Palmer, C.; Zhao, M.; Zhang, Q. Neonicotinoid residues in fruits and vegetables: An integrated dietary exposure assessment approach. *Environ. Sci. Technol.* **2018**, *52*, 3175–3184.
69. Luo, X.; Zeng, X.; Wei, D.; Ma, C.; Li, J.; Guo, X.; Cheng, L.; Mao, Z. Pesticide residues in common fruits and vegetables in Henan Province, China. *Food Addit. Contam. Part B Surveill.* **2023**, *16*, 244–252.
70. Mac Loughlin, T.M.; Peluso, Ma.L.; Etchegoyen, Ma.A.; Alonso, L.L.; de Castro, Ma.C.; Percudani, Ma.C.; Marino, D.J.G. Pesticide residues in fruits and vegetables of the argentine domestic market: Occurrence and quality. *Food Control* **2018**, *93*, 129–138.
71. Mahdavi, V.; Eslami, Z.; Molaee-Aghaee, E.; Peivasteh-Roudsari, L.; Sadighara, P.; Thai, V.N.; Fakhri, Y.; Ravanlou, A.A. Evaluation of pesticide residues and risk assessment in apple and grape from western Azerbaijan Province of Iran. *Environ. Res.* **2022**, *203*, 111882.
72. Matta, D.; Pehme, S.; Peetsmann, E.; Luik, A.; Meremäe, K. Pesticide residues in Estonian local and imported food in 2008–2011. *Acta Agric. Scand. - B Soil Plant Sci.* **2013**, *63*, 78–84.
73. Matyaszek, A.; Szpyrka, E.; Podbielska, M.; Słowik-Borowiec, M.; Kurdziel, A. Pesticide residues in berries harvested from south-eastern Poland (2009–2011). *Rocz. Panstw. Zakl. Hig.* **2013**, *64*, 25–29.
74. Mebdoua, S.; Lazali, M.; Ounane, S.M.; Tellah, S.; Nabi, F.; Ounane, G. Evaluation of pesticide residues in fruits and vegetables from Algeria. *Food Addit. Contam. Part B Surveill.* **2017**, *10*, 91–98.
75. Mert, A.; Qi, A.; Bygrave, A.; Stotz, H.U. Trends of pesticide residues in foods imported to the United Kingdom from 2000 to 2020. *Food Control* **2022**, *133*, 108616.
76. Montiel-León, J.M.; Duy, S.V.; Munoz, G.; Verner, M.-A.; Hendawi, M.Y.; Moya, H.; Amyot, M.; Sauvé, S. Occurrence of pesticides in fruits and vegetables from organic and conventional agriculture by QuEChERS extraction liquid chromatography tandem mass spectrometry. *Food Control* **2019**, *104*, 74–82.
77. Mutengwe, M.T.; Chidamba, L.; Korsten, L. Monitoring pesticide residues in fruits and vegetables at two of the biggest fresh produce markets in Africa. *J. Food Prot.* **2016**, *79*, 1938–1945.
78. Mutengwe, M.T.; Chidamba, L.; Korsten, L. Pesticide residue monitoring on South African fresh produce exported over a 6-year period. *J. Food Prot.* **2016**, *79*, 1759–1766.
79. Mutengwe, M.T.; Aneck-Hahn, N.H.; Korsten, L.; van Zijl, M.C.; de Jager, C. Pesticide residues and estrogenic activity in fruit and vegetables sampled from major fresh produce markets in South Africa. *Food Addit. Contam. Part A Chem. Anal. Control Expo. Risk Assess.* **2016**, *33*, 95–104.
80. Mwanja, M.; Jacobs, C.; Mbewe, A.R.; Munyinda, N.S. Assessment of pesticide residue levels among locally produced fruits and vegetables in Monze district, Zambia. *Int. J. Food Contam.* **2017**, *4*, 11.
81. Nasreddine, L.; Rehaime, M.; Kassaify, Z.; Rechmany, R.; Jaber, F. Dietary exposure to pesticide residues from foods of plant origin and drinks in Lebanon. *Environ. Monit. Assess.* **2016**, *188*, 485.
82. Omeje, J.S.; Asegbeloyin, J.N.; Ihedioha, J.N.; Ekere, N.R.; Ochonogor, A.E.; Abugu, H.O.; Alum, O.L. Monitoring of pesticide residues in fresh fruits and vegetables available in Nigerian markets and assessment of their associated health risks. *Environ. Monit. Assess.* **2022**, *194*, 516.
83. Osaili, T.M.; Al Sallagi, M.S.; Dhanasekaran, D.K.; Bani Odeh, W.A.M.; Al Ali, H.J.; Al Ali, A.A.S.A.; Ismail, L.C.; Mehri, K.O.A.l.; Pisharath, V.A.; Holley, R.; Obaid, R.S. Pesticide residues in fresh fruits imported into the United Arab Emirates. *Heliyon*, **2022**, *8*, e11946.
84. Oshatunberu, M.A.; Oladimeji, A.; Henry, S.O.; Olaniyan, O.A.; Raimi, M.O. Concentrations of pesticides residues in grain sold at selected markets of Southwest Nigeria. *Nat. Resour. Human Health* **2023**, *3*, 387–402.
85. Park, B.K.; Kwon, S.H.; Yeom, M.S.; Joo, K.S.; Heo, M.J. Detection of pesticide residues and risk assessment from the local fruits and vegetables in Incheon, Korea. *Sci. Rep.* **2022**, *12*, 9613.
86. Parveen, Z.R.; Iqbal, S.; Khuhro, M.I.; Bhutto, M.A.; Ahmed, M. Monitoring of multiple pesticide residues in some fruits in Karachi, Pakistan. *Pak. J. Bot.* **2011**, *43*, 1915–1918.
87. Patiño, M.; Valencia-Guerrero, M.F.; Barbosa-Ángel, E.S.; Martínez-Cordón, M.J.; Donado-Godoy, P. Evaluation of chemical and microbiological contaminants in fresh fruits and vegetables from peasant markets in Cundinamarca, Colombia. *J. Food Prot.* **2020**, *83*, 1726–1737.
88. Picó, Y.; El-Sheikh, M.A.; Alfarhan, A.H.; Barceló, D. Target vs non-target analysis to determine pesticide residues in fruits from Saudi Arabia and influence in potential risk associated with exposure. *Food. Chem. Toxicol.* **2018**, *111*, 53–63.

89. Pirsaheb, M.; Fattahi, N.; Rahimi, R.; Sharafi, K.; Ghaffari, H.R. Evaluation of abamectin, diazinon and chlorpyrifos pesticide residues in apple product of Mahabad region gardens: Iran in 2014. *Food Chem*. **2017**, *231*, 148–155.
90. Poulsen, M.E.; Andersen, J.H.; Petersen, A.; Jensen, B.H. Results from the Danish monitoring programme for pesticide residues from the period 2004–2011. *Food Control* **2017**, *74*, 25–33.
91. Qin, G.; Chen, Y.; He, F.; Yang, B.; Zou, K.; Shen, N.; Zuo, B.; Liu, R.; Zhang, W.; Li, Y. Risk assessment of fungicide pesticide residues in vegetables and fruits in the mid-western region of China. *J. Food Compos. Anal*. **2021**, *95*, 103663.
92. Radulović, J.; Lučić, M.; Nešić, A.; Onjia, A. Multivariate assessment and risk ranking of pesticide residues in citrus fruits. *Foods* **2023**, *12*, 2454.
93. Shin, J.m.; Choi, S.-J.; Park, Y.h.; Kwak, B.; Moon, S.H.; Yoon, Y.T.; Jo, S.A.; Yi, H.; Kim, S.j.; Park. S.K.; Park, J.s. Comparison of QuEChERS and Liquid–Liquid extraction methods for the simultaneous analysis of pesticide residues using LC-MS/MS. *Food Control* **2022**, *141*, 109202.
94. Si, W.-S.; Wang, S.-Y.; Zhang, Y.-D.; Kong, C.; Bai, B. Pesticides and risk assessment in Shanghai fruit and raw eaten vegetables. *Food Addit. Contam. Part B Surveill*. **2021**, *14*, 245–255.
95. Sivaperumal, P.; Anand, P.; Riddhi, L. Rapid determination of pesticide residues in fruits and vegetables, using ultra-high-performance liquid chromatography/time-of-flight mass spectrometry. *Food Chem*. **2015**, *168*, 356–365.
96. Skretteberg, L.G.; Lyrån, B.; Holen, B.; Jansson, A.; Fohgelberg, P.; Siivinen, K.; Andersen, J.H.; Jensen, B.H. Pesticide residues in food of plant origin from Southeast Asia – A Nordic project. *Food Control* **2015**, *51*, 225–235.
97. Sójka, M.; Miszczak, A.; Sikorski, P.; Zagibajło, K.; Karlińska, E.; Kosmala. M. Pesticide residue levels in strawberry processing by-products that are rich in ellagitannins and an assessment of their dietary risk to consumers. *NFS J*. **2015**, *1*, 31–37.
98. Soydan, D.K.; Turgut, N.; Yalçın, M.; Turgut, C.; Karakuş, P.B.K. Evaluation of pesticide residues in fruits and vegetables from the Aegean region of Turkey and assessment of risk to consumers. *Environ. Sci. Pollut. Res*. **2021**, *28*, 27511–27519.
99. Suárez-Jacobo, A.; Alcantar-Rosales, V.M.; Alonso-Segura, D.; Heras-Ramírez, M.; E-De La Rosa, D.; Lugo-Melchor, O.; Gaspar-Ramirez, O. Pesticide residues in orange fruit from citrus orchards in Nuevo Leon State, Mexico. *Food Addit. Contam. Part B Surveill*. **2017**, *10*, 192–199.
100. Sungur, Ş.; Tunur, Ç. Investigation of pesticide residues in vegetables and fruits grown in various regions of Hatay, Turkey. *Food Addit. Contam. Part B Surveill*. **2012**, 5, 265–267.
101. Szpyrka, E.; Kurdziel, A.; Matyaszek, A.; Podbielska, M.; Rupar, J.; Słowik-Borowiec, M. Evaluation of pesticide residues in fruits and vegetables from the region of south-eastern Poland. *Food Control* **2015**, *48*, 137–142.
102. Toptanci, İ.; Kiralan, M.; Ramadan, M.F. Levels of pesticide residues in fruits and vegetables in the Turkish domestic markets. Environ. *Sci. Pollut. Res. Int*. **2021**, *28*, 39451–39457.
103. Walorczyk, S.; Drożdżyński, D.; Kowalska, J.; Remlein-Starosta, D.; Ziółkowski, A.; Przewoźniak, M.; Gnusowski, B. Pesticide residues determination in Polish organic crops in 2007–2010 applying gas chromatography–tandem quadrupole mass spectrometry. *Food Chem*. **2013**, *139*, 482–487.
104. Wang, D.; Yang, G.; Yun, X.; Luo, T.; Guo, H.; Pan, L.; Du, W.; Wang, Y.; Wang, Q.; Wang, P.; Zhang, Q.; Li, Y.; Lin, N. Carbendazim residue in plant-based foods in China: Consecutive surveys from 2011 to 2020. *Environ. Sci. Ecotech*. **2024**, *17*, 100301.
105. Wang, S.; Zeng, X.; Wang, X.; Chang, H.; Sun, H.; Liu, Y. A survey of multiple pesticide residues on litchi: A special fruit. *Microchem. J*. **2022**, *175*, 107175.
106. Witczak, A.; Pohoryło, A.; Abdel-Gawad, H.; Cybulski, J. Residues of some organophosphorus pesticides on and in fruits and vegetables available in Poland, an assessment based on the European union regulations and health assessment for human populations. *Phosphorus Sulfur Silicon Relat. Elem*. **2018**, *193*, 711–720.
107. Wołejko, E.; Łozowicka, B.; Kaczyński, P. Pesticide residues in berries fruits and juices and the potential risk for consumers. *Desalin. Water Treat*. **2014**, *52*, 3804–3818.
108. Xing, L.; Wang, Y.; Luo, R.; Li, X.; Zou, L. Determination of 31 pesticide residues in wolfberry by LC-MS/MS and dietary risk assessment of wolfberry consumption. *Food Sci. Technol*, *Campinas* **2022**, *42*, e61921.

109. Yang, G.; Wang, W.; Liang, S.; Yu, Y.; Zhao, H.; Wang, Q.; Qian, Y. Pesticide residues in bayberry (Myrica rubra) and probabilistic risk assessment for consumers in Zhejiang, China. *J. Integr. Agric*. **2017**, *16*, 2101–2109.
110. Yang, X.; Luo, J.; Duan, Y.; Li, S.; Liu, C. Simultaneous analysis of multiple pesticide residues in minor fruits by ultrahigh-performance liquid chromatography/hybrid quadrupole time-of-fight mass spectrometry. *Food Chem*. **2018**, *241*, 188–198.
111. Yang, X.; Luo, J.; Li, S.; Liu, C. Evaluation of nine pesticide residues in three minor tropical fruits from southern China. *Food Control* **2016**, *60*, 677–682.
112. Zhang, Y.; Li, Z.; Jiao, B.; Zhao, Q.; Wang, C.; Cui, Y.; He, Y.; Li, J. Determination, quality, and health assessment of pesticide residues in kumquat in China. *Foods* **2023**, *12*, 3423.
113. Zhang, Y.D.; Si, W.S.; Chen, L.; Shen, G.Q.; Bai, B.; Zhou, C.Y. Determination and dietary risk assessment of 284 pesticide residues in local fruit cultivars in Shanghai, China. *Sci. Rep*. **2021**, *11*, 9681.
114. Zhao, H.; Li, R.; Hu, J. Frequently used pesticides and their metabolites residues in apple and apple juice from markets across China: Occurrence and health risk assessment. *LWT - Food Sci. Technol*. **2023**, *178*, 114610.
